# Complex of Rutin with β-Cyclodextrin as Potential Delivery System

**DOI:** 10.1371/journal.pone.0120858

**Published:** 2015-03-30

**Authors:** Magdalena Paczkowska, Mikołaj Mizera, Hanna Piotrowska, Daria Szymanowska-Powałowska, Kornelia Lewandowska, Joanna Goscianska, Robert Pietrzak, Waldemar Bednarski, Zbigniew Majka, Judyta Cielecka-Piontek

**Affiliations:** 1 Department of Pharmaceutical Chemistry, Faculty of Pharmacy, Poznan University of Medical Sciences, Poznan, Poland; 2 Department of Toxicology, Faculty of Pharmacy, Poznan University of Medical Sciences, Poznan, Poland; 3 Department of Biotechnology and Food Microbiology, Poznan University of Life Sciences, Poznan, Poland; 4 Department of Molecular Crystals, Institute of Molecular Physics, Polish Academy Sciences, Poznan, Poland; 5 Faculty of Chemistry, Adam Mickiewicz University in Poznan, Poznan, Poland; 6 Department of Solid State Radiospectroscopy, Institute of Molecular Physics, Polish Academy Sciences, Poznan, Poland; 7 Adamed Sp. z o.o., Czosnów near Warsaw, Poland; University of Quebect at Trois-Rivieres, CANADA

## Abstract

This study aimed to obtain and characterize an RU-β-CD complex in the context of investigating the possibility of changes in the solubility, stability, antioxidative and microbiological activity as well as permeability of complexated rutin as against its free form. The formation of the RU-β-CD complex via a co-grinding technique was confirmed by using DSC, SEM, FT-IR and Raman spectroscopy, and its geometry was assessed through molecular modeling. It was found that the stability and solubility of the so-obtained complex were greater compared to the free form; however, a slight decrease was observed inits antibacterial potency. An examination of changes in the EPR spectra of thecomplex excluded any reducing effect of complexation on the antioxidative activity of rutin. Considering the prospect of preformulation studies involving RU-β-CD complexes, of significance is also the observed possibility of prolongedly releasing rutin from the complex at a constant level over along period of 20 h, and the fact that twice as much complexated rutin was able topermeate compared to its free form.

## Introduction

Rutin (quercetin-3-rhamnosyl glycoside, RU) is a flavonoid obtained from the flowers of the plant *Ruta officinalis* [1]. The compound is the most common flavonoid contained in pharmaceutical preparations and dietary supplements due to its broad spectrum of pharmacological activity including anti-inflammatory, antimicrobial, antioxidant, asthma-reducing and cholesterol-lowering actions as well as preventive potency against neuronal disease and cancer [2–8]. Those advantages, however, may be offset by the low effectiveness of rutin or its absorbability from the alimentary tract as a result of the compound's poor solubility, susceptibility to degradation and low bioavailability. Consequently, there are efforts to improve the solubility, chemical stability and absorbability of rutin. The most widely applied solutions include chemical modification aimed at receiving derivatives with better dissolving properties (e.g., a diacetylated derivative used in eye drops for prophylaxis in diabetic retinopathy) and sugar coating to reduce rutin degradability in solid-phase drug forms prone to UV radiation [9]. The poor solubility and resultant low bioavailability of rutin have stimulated research into the possibility of combining the flavonoid with a modifier. Enhanced dissolution and absorption of biologically active substances may be achieved by creating complexes with cyclodextrins, which meet all the requirements for up-to-date pharmaceutical modifiers. Cyclodextrins (CDs) are oligosaccharides with hydrophilic external surface and hydropholic internal cavity. CDs can act as complexing compounds that increase aqueous solubility of hydrophilic drug, enhance permeability and adsorption of drug across membrane barrier drugs and improve chemical stability of labile drugs [10–12].

According to A.N. Nguyen, the complexation of rutin with cyclodextrins is a spontaneous exothermic reaction described by negative values of the enthalpy change (ΔH). Inclusion complexation with β-hydroxypropyl-cyclodextrins (β-HP-CD) has been found to have the greatest stabilizing effect on rutin in aqueous solutions exposed to various stress factors allowing the compound to retain its anti-oxidant activity [13]. The insertion of the A ring of rutin into the cavity of β-HP-CD has been suggested to be responsible for its supramolecular interactions with the cyclodextrin. Although, a few papers mentioned about interactions between RU and cyclodextrins (such as β-CD and methyl-β-CD) [14–16], the reports on the complexation of rutin with cyclodextrins are not paralleled by any communications about the possibilityadvantages of modifying the properties of rutin through its complexation. Zhao J. reported kinetic and thermodynamic studies of rutin absorption with β-cyclodextrin polymer adsorbent [17].

The aim of this work was to analyze the possibilityadvantages of modifying properties ofrutinsuchas chemical stability, solubility, antibacterial activity, dissolvability and permeability, which are important for the therapeutic safety and effectiveness of the compound, by its complexation with β-cyclodextrins (β-CD).

## Materials and Methods

### 2.1 Materials

Rutin and its related substances (isoquercetin, quercetin), β-cyclodextrin (purity > 98%) were supplied by Sigma-Aldrich (Poland). Acetonitrile of an UHPLC grade was supplied by Merck KGaA (Germany) and formic acid (100%) by P.O.Ch. (Poland). High-quality pure water was prepared using an Exil SA 67120 Millipore purification system (France). All other chemical were of analytical reagent grade.

### 2.2 Preparation of the RU-β-CD inclusion complex

The complex was obtained by kneading rutin with β-CD in the same molar ratio 1: 1 with continuous stirring for 30 min. Then the RU-β-CD complex was kept at313 K in ambient relative humidity.

### 2.3. Characterization of the RU-β-CD inclusion complex

#### 2.3.1. FT-IR spectroscopy (FT-IR)

RU, β-CD andRU-β-CD inclusion complex co-grind mixture were obtained separately with IR grade KBr in the ratio of 1: 100, and corresponding pellets were prepared by applying 8 metric ton of pressure in hydraulic press. The vibrational infrared spectra were recorded between 400 and 5000 cm^-1^, with an FT-IR Bruker Equinox 55 spectrometer equipped with a Bruker Hyperion 1000 microscope. In order to analyse changes in positions and intensity in experimental spectra of RU-β-CD inclusion complex, quantum chemical calculations based on DFT were performed. All the calculations were made by using the Gaussian 03 package [18]. The GaussView application was utilized to propose the initial geometry of the investigated molecules and to visually inspect the normal modes.

#### 2.3.2. UV spectroscopy

An UV-VIS Lambda 20 (Perkin Elmer) spectrophotometer equipped with 1.0 cm-in-width quarts cells and controlled by the UV WinLab software was utilized for the determination of changes of rutin concentration. UV spectroscopy was used during dissolution studies of RU from its complex RU-β-CD.

#### 2.3.3. Differential scanning calorimetry (DSC)

DSC analysis of RU and RU-β-CD inclusion complex were performed by using differential scanning calorimeter (Mettler Toledo DSC 1 Star System, Zurich, Switzerland). The cell constant calibration method was applied to analysis the DSC patterns of the samples form from 313 K to 613 Kat a heating step 10 K, under a constant flow 10 K/min ofnitrogen atmosphere.

#### 2.3.4. Scanning electron microscopy (SEM)

Prior to the study all samples were coated with mixture of gold and palladium in Polaron Range SC7620 Sputter Coater. Time of coating was set to 135 seconds. The surfaces of RU, β-CD, RU-β-CD were observed with the use of Hitachi S-3000N Scanning Electron Microscope.

#### 2.3.5. EPR spectroscopy

Detection of free radicals and determination of their concentration were carried out using a Bruker ELEXSYS 500 spectrometer (X-band) at room temperature. EPR spectra were recorded as a first derivative of the absorption signal. The number of free radicals was calculated using the double integration procedure described elsewhere [19].

#### 2.3.6. UHPLC-DAD chromatography

The LC system (DionexThermoline Fisher Scientific, Germany) was equipped with a high pressure pump (UltiMate 3000), an autosampler (UltiMate 3000) and a DAD detector (UltiMate 3000). For data processing and acquisition, Chromeleon software version 7.0 from Dionex Thermoline Fisher Scientific (US) was used. As the stationary phase, a Kinetex C-18 column, 1.7 μm particle size, 100 mm x 2.10 mm (Shim-Pol, Poland) was used. The mobile phase consisted of acetonitrile–0.1% formic acid (20:80 *V/V*). The flow rate of the mobile phase was 0.4 mL/min. The wavelength of the UV detector was set at 353 nm. The injection volume was 5.0 μL. The changes of concentration of rutin by using the UHPLC-DAD method was used during stability studies, phase solubility studies and permeability studies.

#### 2.3.7. Theoretical studies

To determine the way of inclusions of rutin into β-CD, docking software AutoDockVina was used. Conformations with the lowest binding energy were subjected to optimization using molecular modeling MM2. The layered model calculations ONIOM was used for exact conformation of the complex RU-β-CD. The GaussView software was utilized to propose the initial geometry of the investigated molecules and to visually inspect the normal modes. The molecular geometries were optimized by means of a density functional theory (DFT) method with the B3LYP hybrid functional and a 6-31G(d,p) basis set [18].

### 2.4. Studies of RU-β-CD inclusion complex

#### 2.4.1. Monitoring the radials of RU-β-CD inclusion complex

The measurement of radicals in non-degraded samples of RU and samples of RU-β-CD which were susceptible to exposition to light (1.2 × 10^-6^ Lux/h) for 24 h was studied by using EPR spectroscopy and compared to the radicals level in free rutin.

#### 2.4.2. Stability studies of RU-β-CD inclusion complex

The degradation of RU-β-CD was studied in aqueous solutions: in hydrochloric acid (c = 0.5 mol/L), sodium hydroxide (c = 0.2 mol/L) and in hydrogen peroxide (25%). While the degradation of rutin in the solid state was studies at as the effect of exposition to light (1.2 × 10^-6^ Lux/h). Photodegradation stability studies of rutin in the solid state were performed using Suntest CPS^+^ (Atlas) with Solar ID65 filter. The cells were exposed to UV radiation (300–400 nm) in an overall illumination of ≥ 210 wh/m^2^ in Suntest C+.

Samples of RU-β-CD for stability studies were prepared by dissolving an accurately weighed 18.0 mg (containing 5.0 mg of free rutin) in 25.0 mL of the equilibrated solution to 298 K in stopped flasks. At specified times, samples of the reaction solutions (1.0 mL) were collected and instantly cooled with a mixture of ice and water, neutralized with 0.8 mL of NaOH or HCl solutions of suitable concentrations and assayed. The ionic strength of all solutions was adjusted to 0.5 mol/L with a solution of sodium chloride (4 mol/L). At specified time intervals, determined by the rate of degradation, the samples of RU-β-CD (5.0 mg) were removed, cooled to room temperature and their contents were dissolved in 5.0 mL of methanol. The so-obtained solutions were quantitatively transferred into measuring flasks and diluted with water to 25.0 mL and chromatographic determination was conducted.In order to check the influence of temperature on degradation in HCl (c = 0.5 mol/L) samples of rutin were degraded at 323, 333, 343, 353 K; while its degradation in solution of NaOH (c = 0.2 mol/L) was performed at 303, 308, 313 and 318 K.

#### 2.4.3. Phase solubility studies of RU-β-CD inclusion complex

Phase-solubility profile of RU-β-CD complex was determined according to the method of Higuchi and Connors [20]. The excess amount of rutin (1.0–50.0 mg) was added to samples of β-CD, with the concentration from the range of rutin concentration. Samples containing RU-β-CD mixture were shaken for 1 h at 298 K. The suspensions were filtered through cellulose membrane filters (0.45 μm). The concentrations of rutin were determined by using UHPLC-DAD method. The phase-solubility of rutin were obtained by plotting the solubility of rutin as the function of concentration of β-CD. Because stechiometric balance between RU and β-CD was 1:1, the following equation was applied to establish apparent stability constant of RU-β-CD:

$${\text{K}}_{1:1}=\frac{\text{slope}}{{\text{S}}_{0}\left(1-\text{slope}\right)}$$ (1)

where S_0_ is the intercept and slope is angular coefficient of the fitted straight line.

#### 2.4.4. Microbiological activity of RU-β-CD inclusion complex

The antibacterial activity of RU-β-CD complex was tested *in vitro* against a selection of indicator bacteria strainsby determining the minimum inhibitory concentration (MIC) as well as analyzing the kinetics of changes in the concentrations of various bacteria caused by the action of RU-β-CD complex. Strains from bacterial strain bank as well as clinical isolates were used in the study. Cultures of *Pseudomonas aeruginosa*, *Listeria monocytogenes*, *Salmonella enteritidis*, *Staphylococcus aureus* and *Escherichia coli* were cultured in soy-caseine broth with yeast extract for microorganisms with increased nutritional requirements. *Clostridium butyricum* and *Clostridium pasterianum* were grown in Reinforced Clostridial Medium (RCM, Oxoid, UK). The strains came from the collection of the Department of Biotechnology and Food Microbiology, University of Life Sciences, Poznan, Poland and American Type Culture Collection. MICs were determined in liquid medium using successive dilutions method. The concentrations of the RU and RU-β-CD complex solutions were lowered to 0.02–250 mg/L. First, test tubes with liquid medium for bacteria were prepared. Then RU and RU-β-CD complex solutions of decreasing concentrations were added to each of test tubes. Next, test tubes were inoculated with the same amount of cells suspension. After 16–18 hours of incubation in 310 K the growth of strains was checked via turbidity increase observation. In test tubes containing less then MIC of examined drugs the turbidity increase was observed (the cells have grown). The minimal concentration of drug/s that inhibited strain growth was defined as MIC.

#### 2.4.5. Dissolution profile of RU-β-CD inclusion complex

Dissolution studies of rutin from RU-β-CD complex were performed by using Vankel diffusion apparatus 7010 connected to the UV-VIS spectrophotometer Cary 50 Bio (Varian). In the first step the samples were placed in the extraction chamber and then covered with a porous synthetic mesh to maintain a constant active substance release surface. So-prepared chambers were introduced into the beaker filled with mixture of ethanol-water (20:80 *V/V*). Tests were performed for 48h at pH 7.2 at 310 K ± 0.5 K while maintaining the agitation rate was 100 rpm. At appropriate time intervals, samples were collected automatically and spectrophotometric analysis was performed at a wavelength suitable for rutin λ_max_ = 256 nm.

Based on the obtained results, the amount of released active substance was calculated by using the following equation:

$$\%\text{released}=\;\left(\frac{{\text{A}}_{\text{p}}}{{\text{A}}_{\text{w}}}\right)\left(\frac{{\text{m}}_{\text{w}}\left[\text{mg}\right]\cdot {\text{C}}_{\text{w}}}{{\text{V}}_{\text{w}}\left[\text{mL}\right]}\right)\left(\frac{1}{{\text{D}}_{\text{w}}}\right)\left(\frac{{\text{V}}_{\text{p}}\left[\text{mL}\right]}{{\text{m}}_{\text{p}}\left[\text{mg}\right]}\right)\cdot 100\text{\%}$$ (2)

where: A_p_—absorbance of the sample, A_w_—absorbance of the standard, m_w_—mass of the standard, m_p_—mass of the rutin contained in the sample, C_w_—cleanliness of the standard, D_w_—dilution of the standard, V_w_—volume of the standard solution, V_p_—volume of acceptor fluid.

#### 2.4.6. Permeability studies of RU-β-CD inclusion complex

CaCo-2 colon cancer cell line was purchased from the European Type Culture Collection. CaCo-2 cell line was maintained in phenol red-free DMEM supplemented with 10% foetal bovine serum (FBS), 2 mmol/L glutamine, penicillin (100 U/mL), and streptomycin (0.1 mg/mL). Cells were cultivated under standard conditions at 310 K in a humidified atmosphere containing 5% CO_2_ and 95% air. For transport assay, the CaCo-2 cells were seeded at 4x10^5^ cells/cm^2^ onto transparent membranes (Millipore) (pore size 0.4 μm, growth area 0.6 cm^2^) in clusters of 12 wells (Falcon). The growth medium was changed three times a week until time of use. Permeability of RU and RU-β-CD was measured under iso-pH condition (pH 7.4A–7.4B) in the apical-to-basolateral (A-B) and basolateral-to-apical (B-A) directions. Before initiation of the transport studies and after the test, TEER (trans epithelial electric resistance) was measured with Millicell ERS-2 Epithelial Volt-Ohm Meter. Only cells with TEER values of >450 Ω were used for the assay. Test compounds were diluted in HBSS buffer (pH 7.4) at concentration of 5 mg/mL. The volume on the apical side was maintained at 400 μL and the volume on the basolateral side was 600 μL. Samples were taken from the receiver and donor side at 15, 30, 60, 90, 120 min, replace with HBSS and run in triplicate.

## Results and Discussion

The first part of the work involved preparing the RU-β-CD complex. In the second part, the so-obtained complex was studied with respect to the possibility of modifying the chemical stability, solubility, antibacterial and antioxidant (free radicals quenching) activity, dissolvability and permeability of rutin. The results received for the RU-β-CD complex were compared with those obtained for free rutin.

The RU-β-CD inclusion complex was obtained via grinding technique. The formation of complex was spontaneous process and repeatable. An apparent stability constant of RU-β-CD was 234 mmol/L. From 0.2 mg/mL, the rate of formation of RU-β-CD was constant, what it is important for repeatability of future preformulation process with the presence of RU-β-CD complex. The formation of RU-β-CD complex was characterized by FT-IR, DSC and SEM. A geometry of RU-β-CD complex was established by theoretical calculations.

**Fig 1:**
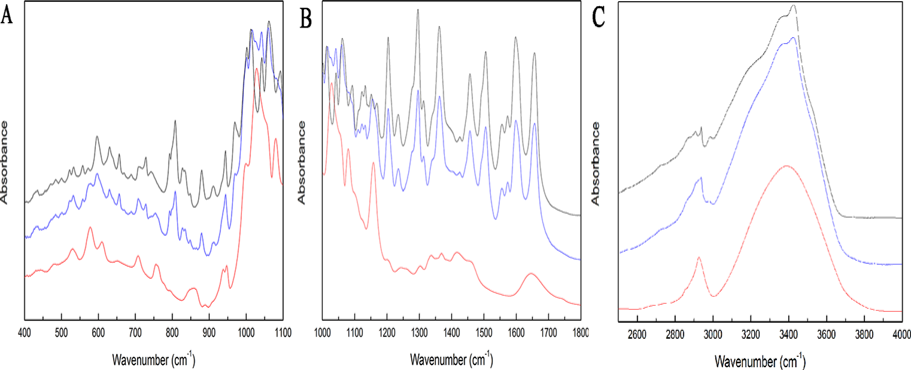
FT-IR absorption spectra for β-CD (red), RU (black) and RU-β-CD complex (blue).

FT-IR spectra of RU (red line), β-CD (black line) and RU-β-CD inclusion complex (blue line) are displayed in Fig. 1. Based on computational methods with usage of the 6-31G(d,p)++ basis set, characterized bands confirmed formation of RU-β-CD complex were identified in experimental spectra. The most intensive differences in positions, intensity and width of the ranges: 500–1200 cm^-1^ and about 3388 cm^-1^ were recorded for RU-β-CD complex in comparison to RU and β-CD spectra. In the region 500–1200 cm^-1^, the strong bands associated with the stretching vibration of the C-C, C-O, and wagging vibration of the C-H bonds in the rutin were also observed. For the β-CD, the quit strong bands at 1029, 1079, and 1157 cm^-1^ related to the stretching vibration of the C-C, C-O bonds, and wagging vibration of the C-H bonds directly at the sugar ring were also recorded. These bonds for RU-β-CD complex were shifted to the 1026, 1083, and 1160 cm^-1^, respectively. Below, at 578, 610, 707, 756, 938, 948 cm^-1^, and wide band at 856 cm^-1^ which were related to the wagging vibration of the O-H bonds directly at the sugar ring, rocking vibration of the O-H bonds, and to the stretching vibration of the C-C and C-O bonds for the β-CD were observed. In FT-IR spectrum of the RU-β-CD these bands were quite different. For the RU-β-CD, the bands at 578, 610 cm^-1^ and additional components at 596 cm^-1^ related to the vibration modes in RU were observed. For the complex the subsequent differences at about 700–750 cm^-1^ were visible, where changes in the relativeintensity ofthe bandsand theirlocation, and shape were observed. Also the band in two maxima at 938 and 948 cm^-1^ for RU-β-CD with one maximum at 943 cm^-1^ was broadened. Whereas the wide band at 856 cm^-1^ for complex was almost completelyinvisible. As the above the range, changes of vibration of bands C-O, C-C-H connected with bonds in dihydroxyphenyl ring were also visible.The extremely band at about 3388 cm^-1^ was associated with the symmetric and antisymmetric O-H stretching modes of the β-CD molecules. While this broad band in the spectrum of RU-β-CD complex distinguished bytwomaxima of the band at 3355 and 3424 cm^-1^, and they are also broadened. The changes may be accounted for in terms of OH stretching modes associated with a β-CD bridged systems and they are in accordance with the literature [21]. In the range 2500–3000 cm^-1^, the changes in the FT-IR absorption spectra for RU-β-CD complex were smaller. In this region the bands associated with the symmetric and antisymmetric stretching vibration of the C-H bond in both RU and β-CD molecules are located and changes can be connected with occurs hydrogen bonding of RU segments in cavity of β-CD.

**Fig 2:**
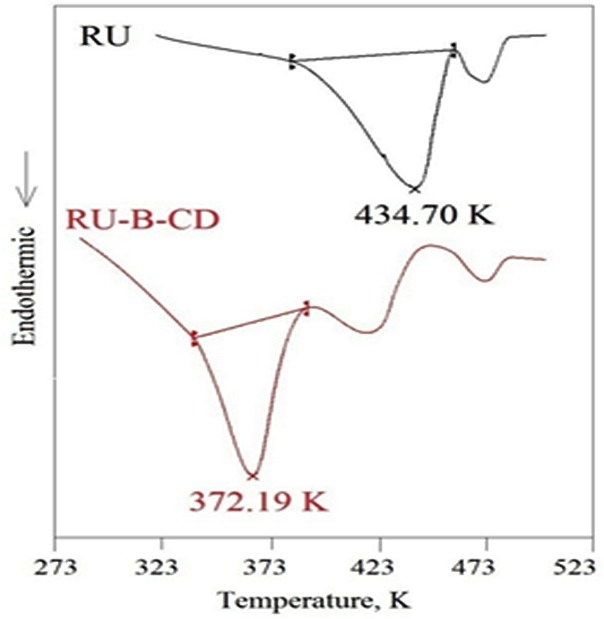
DSC spectra of RU (black) and RU-β-CD (red).

The DSC thermograms of RU (black line), and RU-β-CD inclusion complex (red line) are shown in Fig. 2. β-CD and RU have shown individual endothermic peaks at 503.0 K and at 434.70 K, respectively. Whereas, in the case of RU-β-CD inclusion complex prominent peaksbelonging to RU completely disappeared. The thermogram of RU-β-CD shows a characteristic endothermic peak at 372.19 K. This can be attributed to the RU being included into the β-CD cavity. Lower of melting point of RU-β-CD complex than ones reported for RU and β-CD, it is agreement with the reported result for complex of rutin with β-CD obtained as the result of co-precipitation [13]. It may be suggested that the preparation of RU-β-CD complexes by co-grinding the components in the solid state significantly reduces hydrogen bonding and van der Waals interactions connection with content of water, which accounts for the lower melting point of the so-obtained complexes.

**Fig 3:**
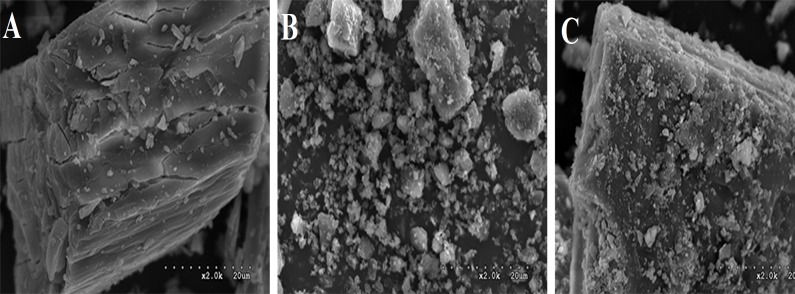
SEM images of β-CD (A), RU (B), RU-β-CD (C).

The overall bulk surface morphology of RU, β-CD and RU-β-CD inclusion complex was studied by using SEM (Fig. 3). Even distribution of RU on β-CD suggests that the inclusion complexation process between β-CD and RU occurred.

**Fig 4:**
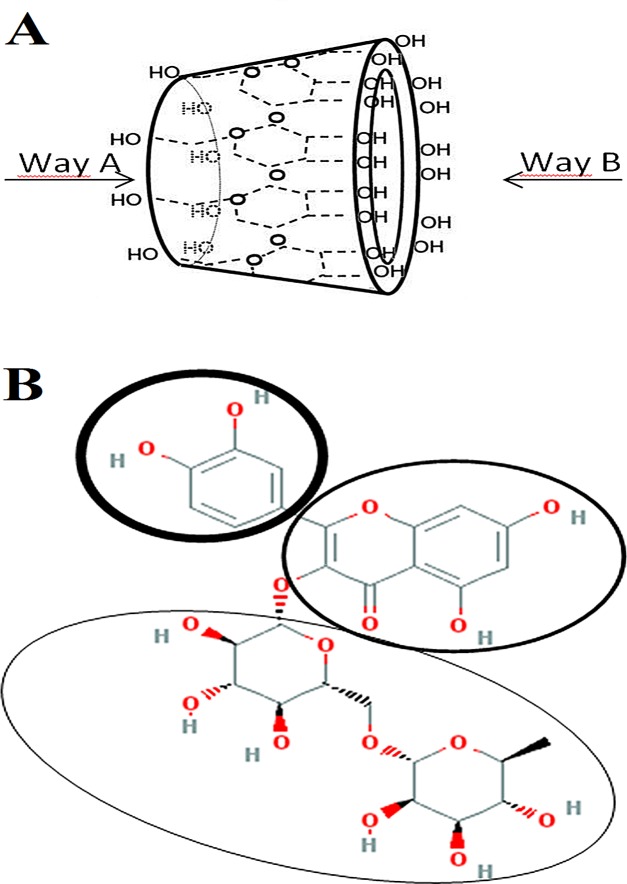
Two possible ways of inclusion of rutin into β-CD (A) and the structural parts of routine which can interact with β-CD during complex creation (B).

Possible mechanism of formation of RU-β-CD inclusion complex was proposed by theoretical calculations. The task of establishing geometry of RU-β-CD inclusion complex can be divided into three parts: determining the most possible way of inclusion, determining the structural part of rutin compound which interacts in non-covalent way with atoms of inner side of β-CD, determining relative position of both molecules in complex. Two possible ways of inclusion were presented on Fig. 4a, while structural parts of rutin which can interact with inner part of β-CD were presented on Fig. 4b. Semi-empirical PM6 molecular modeling was applied in order to determine the most probable relative positions of molecules with accuracy of 15°. PM6 method allows handling hydrogen bonds better than MM2 method. It is crucial to capture such interactions in RU-β-CD complex to optimize geometry properly. Initial structures for semi-empirical modeling were established by rotation of rutin in horizontal axis relatively to β-CD molecule in complex geometries acquired by MM2 modeling. Each of rotated structures was optimized with PM6 method. Binding energy of rutin and β-CD was measured for each rotated complex according to formula:

ΔE=E(BCD⋯R)−[E(BCD)+E(R)] (3)

where, ΔE is the binding energy, E(BCD⋯R) is the energy of complex, [E(BCD)+E(R)] is the sum of energies of free β-CD and rutin molecules. The structure with the lowest binding energy was considered the best one.

**Fig 5:**
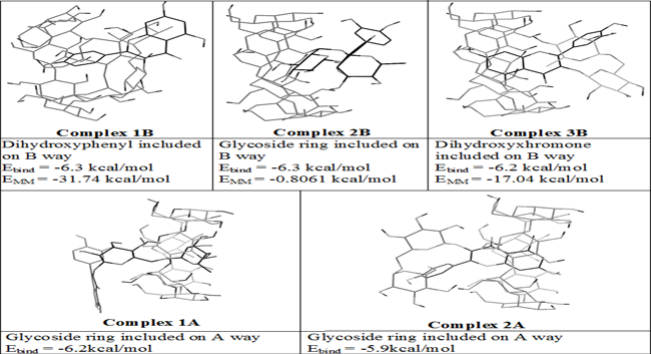
Binding modes of β-CD and RU-β-CD inclusion complex.

**Fig 6:**
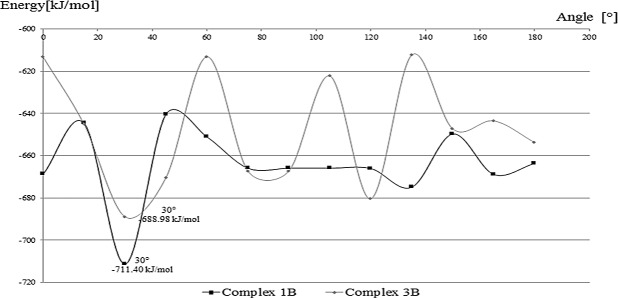
Changes of binding energy of molecules in complex in respect to rotation degree.

The AutoDockVina docking program appointed five of the most probable configuration of structure of the inclusion complex. Complex geometries are presented in Fig. 5. Probability of creation of a durable bond between RU and β-CD was determined based on the estimated affinity energy E_bind_. For the designated conformation, the highest affinity energy characterized complexes formed by inclusion bythe way B. These complexes were subjected to geometry optimization using molecular modeling. Binding energies E_MM_ of complexes molecules modeled with MM2 method are shown in Fig. 5. Further examination with semi-empirical calculations confirmed results obtained with molecular mechanics and allowed to determine the optimal position relative to the rutin of β-CD molecule. Studies were carried out for complexes which are the most energetically favored: 1B and 3B. Geometry of 1B complex modified by rutin rotation of 30 degrees in the horizontal plane is considered as the best of the geometry of the structures tested. The geometry resulted from semi-empirical optimization of the structures are shown in Fig. 6.

The second part of work aimed studies of modification of properties of rutin after introduction it into cavity of β-CD. Results of studies for RU-β-CD were compared with those obtained for free RU.

**Fig 7:**
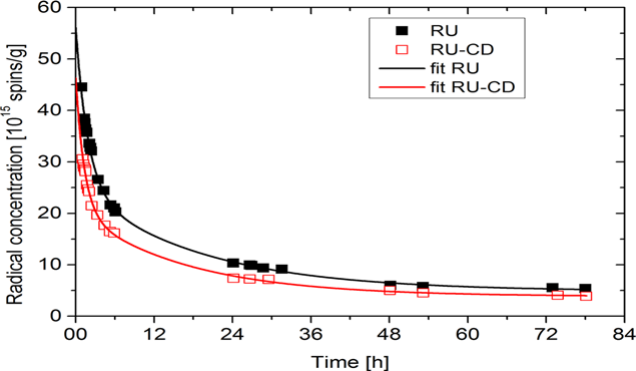
The disappearance of free radicals after irradiation. Approximations to experimental points were done according to equation 4 and appropriate parameters are collected in table 1.

For RU-β-CD inclusion complex, level of radials was lower than in the case of free rutin. It decreasedfrom the value 1.2 (±0.2) × 10^15^ [radicals/g] for free rutin to the value 0.6 (±0.2) × 10^15^ [radicals/g] for RU-β-CD. The process of the disappearance of free radicals after irradiation in the RU and RU-β-CD complex is described by the following equation:

$$I\left(t\right)=I_{1}e^{-\frac{t}{t_{1}}}+I_{2}e^{-\frac{t}{t_{2}}}+I_{0}$$ (4)

where the first and the second term of equation correspond to nonstable radicals and I_0_ is the concentration of stable radicals. I(t) is the free radical concentration at any time t after irradiation, I_1_ is the concentration of radicals disappearing at mean lifetime t_1_ and I_2_ is the concentration of radicals disappearing at mean life time t_2_. Thus, the time at which the level of free radicals after irradiation reached a constant value was determined. The process of free radicals quenching as a function of time follows almost the same route for RU and RU-β-CD complexes, although it is more intensive for the latter (Fig. 7, Table 1).

**Table 1:** Parameters characterizing the concentration of free radicals described by equation 4.

	I(t = 0h)	I_0_	I_1_	t_1_ [h]	I_2_	t_2_ [h]
	[10^15^ radicals/g]	[10^15^ radicals/g]	[10^15^ radicals/g]		[10^15^ radicals/g]	
RU—bulk substance	1.2 (±0.2)	1.2 (±0.2)	-	-	-	-
RU—after exposure to sunlight (24h)	56.2 (±3.0)	4.9 (±0.5)	31.1 (±1.3)	1.87 (±0.21)	20.2 (±1.2)	19.0 (±1.9)
RU-β-CD—0h	0.6 (±0.2)	0.6 (±0.2)	-	-	-	-
RU-β-CD—after exposure to sunlight (24h)	46.3 (±3.0)	3.8 (±0.3)	25.9 (±1.9)	1.34 (±0.17)	16.6 (±0.8)	17.0 (±1.7)

**Fig 8:**
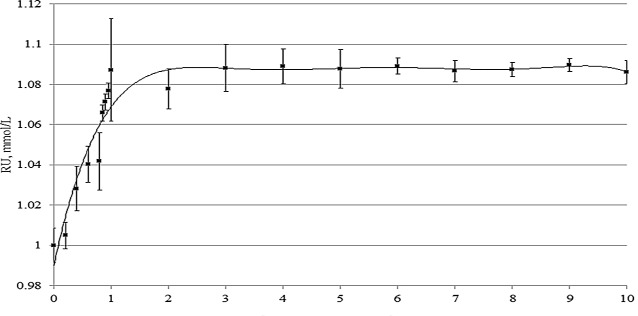
Phase-solubility diagram of RU-β-CD inclusion complex.

The study of changes of solubility of RU after complexation was measured by using UPLC-DAD.The profile of the phase-solubility of RU is presented in Fig. 8. The solubility of RU increases as a function of CD concentration showing a typical A_N_ type profile [10]. The apparent stability constant (K_1:1_) of formation of RU-β-CD complex was calculated from the slope of phase-solubility diagram and it was (0.09269 M^-1^). At a concentration of 1.0mmol/L of β-CD the solubility of RU was increased from 1.0 to 1.09 mmol/L, which represents slightly increase of the solubility of RU in methanol:water. From 3.0 mmol/L of rutin, there was not observed RU solubility increasing with β-CD concentration growth. Obtaining the constant relationship between active substance and carrier it is important for system RU-β-CD as potential drug delivery because the stability of the system affects the reproducibility of processes during preformulation studies with his participation.

According to stability reports, rutin is susceptible to degradation as the effect action of physico-chemical factors [22]. As the result of our stability studies of RU, comparison observed rate constants of rutin degradation in free form and in complex were conducted for its degradation in hydrochloric acid (c = 0.5mol/L, T = 323–353 K), solvent of sodium hydroxide (c = 0.2mol/L, T = 303–318 K), in oxidizing agent (H_2_O_2_, c = 25%, T = 353 K) and during photolysis (1.2 × 10^-6^ Lux/h). The kinetic mechanism of rutin degradation did not change after its complexation. A degradation of rutinwas a pseudo-first-order reaction described by the equation:

$$\ln\left(P_{RU}\right)=\ln{\left(P_{RU}\right)}_{0}-k_{obs}$$ (5)

were *P*_*RU*_ are the areas of the peaks of rutin at time *t* = 0 and *t*, respectively.

**Fig 9:**
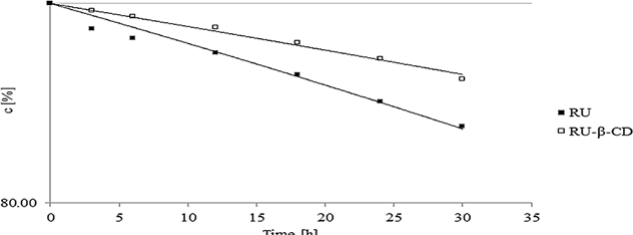
The semi-log plots of c = f(t) for the degradation of RU and RU-β-CD after its exposure to sunlight.

**Fig 10:**
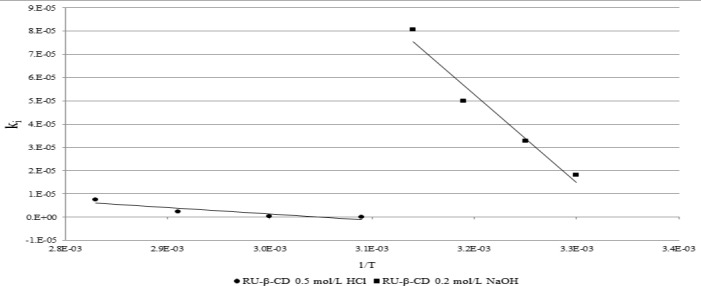
The semilogarithmic relationship k_i_ = f(1/T) for the degradation of RU-β-CD in 0.5 mol/L HCl (●) and in 0.2 mol/L NaOH (■).

The semi-logarithmic plotsln(P_RU_)/(P_RU_)0 × 100 = *f*(t)were linear and their slopes were equal to the rate constants of the reactions with the negative sign (-k_obs_).The differences in profile of formed degradation products for free rutin and for RU-β-CD was not observed. However, comparison of observed rate constantscalculated fordegradation of rutinshows significant statistic differences between their values (Table 2). The most stabilization effect of RU by introduction it into β-CD was observed during photolysis, oxidation and acidic hydrolysis (Fig. 9). To verify that k_obs_ determined for degradation of RU and RU-β-CD were statistically insignificant the parallelism test was used. On completion of the designed degradation experiments with RU-β-CD, the relationship between the reaction rate constants and temperature was described by the Arrhenius equation:

$$lnk_{i}=lnA-E_{a}/RT$$ (6)

where *k*_*i—*_the reaction rate constants of RU from RU-β-CD [s^-1^], *A—*frequency coefficient, *E*_a—_activation energy [J mol^-1^], *R*—the universal gas constant [8.3144 J K^-1^ mol^-1^], and *T*—temperature [K]. The straight-line relationship ln*k*_*i*_ = *f*(1/*T*) was obtained for rutin in the temperature ranges 323–353 K during acid hydrolysis and 303–318 K during basic hydrolysis. The least squares method was used to calculate the slopes (*a*) and the frequency coefficient (ln A), which allowed calculation of activation energy (*E*_*a*_ = −*a* × R, enthalpy (*ΔH* ^≠^) and entropy (*ΔS* ^≠^) at 298 K (Fig. 10, Table 2). The entropy of degradation of RU during acidic and basic hydrolysis as well as for RU-β-CD during basic hydrolysis was negative which may indicate a bimolecular character of degradation (Tables 2). While the kinetic mechanism of degradation of RU-β-CD in acidic hydrolysis was different. The following statistical parameters of the equation y = *a*x + *b* were calculated by using the least squares method: *a±*Δ*a*, *b±*Δ*b*, standard deviations *S*_*a*_, *S*_*b*_, *S*_*y*_ and the coefficient of linear correlation *r*. The values Δ*a* and Δ*b* were calculated for *f* = *n*—2 degrees of freedom and α = 0.05.

**Table 2:** Kinetic and thermodynamic parameters of the degradation of RU and RU-β-CD during acidic-basic hydrolysis.

Temperature, K	RU		RU-β-CD		t_0_
	k_obs_, s^-1^	Thermodynamic parameters	k_obs_, s^-1^	Thermodynamic parameters	
0.5 mol/L HCl					
323	(2.75 ± 0.43) × 10^-7^	*E*_*a*_ = 122.97 ± 30.42 (kJ/mol))	(1.86 ± 5.64) × 10^-7^	*E*_*a*_ = 114.90 ± 30.42 (kJ/mol)	2.7754
333	(6.89 ± 0.42) × 10^-7^	*ΔH* ^≠a^ *=* 120.49 ± 32.90 (kJ/mol)	(3.57 ± 0.24) × 10^-7^	*ΔH* ^≠a^ *=* 112.13 ± 32.90 (kJ/mol)	9.4814
343	(2.47 ± 2.06) × 10^-6^	*ΔS* ^≠a^ *=* -4.10 ± 22.30 (J/Kmol)	(2.25 ± 6.02) × 10^-6^	*ΔS* ^≠a^ = -18.18 ± 22.30 (J/Kmol)	2.7754
353	(8.86 ± 0.44) × 10^-6^		(7.39 ± 0.15) × 10^-6^		4.3675
0.2 mol/L NaOH					
303	(1.81 ± 0.98) × 10^-5^	*E*_a_ = 75.26 ± 30.42 (kJ/mol)	(1.81 ± 0.99) × 10^-5^	*E*_a_ = -29.80 ± 30.42 (kJ/mol)	0
308	(3.27 ± 0.33) × 10^-5^	Δ*H* ^≠a^ = 72.79 ± 32.90 (kJ/mol)	(2.19 ± 0.10) × 10^-5^	Δ*H* ^≠a^ = -30.05 ± 32.90 (kJ/mol)	4.4875
313	(4.99 ± 0.40) × 10^-5^	Δ*S* ^≠a^ = -86.94± 22.30 (J/Kmol)	(4.08 ± 0.49) × 10^-5^	Δ*S* ^≠a^ = -150.88± 22.30 (J/Kmol)	1.9454
318	(8.06 ± 0.02) × 10^-5^		(5.29 ± 0.01) × 10^-5^		1.9064
25% H_2_0_2_					
353	(1.62 ± 0.91) × 10^-6^	-	(1.11 ± 0.83) × 10^-6^	-	5.4930
Photolysis					
-	(1.18 ± 0.91) × 10^-6^	-	(5.57 ± 0.54) × 10^-7^	-	5.9499

*Δk = S*_*a*_ *t*_*α*_ *f E*_*a*_, activation energy; *ΔH* ^≠^, enthalpy; *ΔS* ^≠^, entropy; *Ea = -aR*; *ΔH* ^≠^ *= E*_*a*_ *-TR*; *ΔS* ^≠^ *= R(ln A ln(k*_*b*_ *T)/h* where: k_B_, Boltzmann’s constant (1.3807 10^-23^ JK^-1^); *h*, Planck’s constant (6.626 10^-34^Js); *R*, universal gas constant (8.314 K^-1^mol^-1^), *T*, temperature in K; a, vectorial coefficient of the Arrhenius; *A*, frequency coefficient *a*calculated for 298 K; t_0_, parameter of parallelism test, establishing significance of *a* coefficient of ln(c_i_) = f(t) plots.

Microbiological studies of the effectivity of RU-β-CD complex and RU against Gram-positive and Gram-negative bacteria are collected in Table 3. Among examined model strains, higher inhibitory concentration of RU-β-CD was observed for *Staphylococcus aureus*, *Staphylococcus aureus* ATCC 25923, *Escherichia coli*, *Escherichia coli* ATCC 25922 than in the case of RU.While the RU-β-CD shown the 2-fold higher antimicrobiogical activity against *Pseudomonas aeruginosa* (MIC = 5000 mg/L *vs*. MIC = 10 000 mg/L for RU and RU-β-CD, respectively), *Pseudomonas aeruginosa* ATCC 27853 (MIC = 5000 mg/L *vs*. MIC = 10 000 mg/L for RU *vs*. RU-β-CD, respectively) and *Proteus vulgaris* (MIC = 5000 mg/L *vs*. MIC = 10 000 mg/L for RU and RU-β-CD, respectively). The effect of microbiological studies permitted to suggest the influence of system RU-β-CD on the efflux system, which result could be better activity of RU-β-CD against bacteria, which involved the process during induction of resistance.

**Table 3:** MIC (mg/L) of RU and RU-β-CD inclusion complex against selected Gram-positive and Gram-negative bacteria.

Microorganism	RU	RU-β-CD
The clinical isolate or indicator		inclusion
		complex
	mg/L	
*Salmonella enteritidis*	NF**	NF
*Salmonella enteritidis ATCC 13076*	NF	NF
*Listeria monocytogenes*	NF	NF
*Listeria monocytogenes ATCC 7644*	NF	NF
*Staphylococcus aureus*	5000	10.000
*Staphylococcus aureus ATCC 25923*	3125	6250
*Escherichia coli*	6250	12500
*Escherichia coli ATCC 25922*	12500	25000
***Pseudomonas aeruginosa***	**10.000**	**5000**
***Pseudomonas aeruginosa ATCC 27853***	**10.000**	**5000**
***Proteus vulgaris***	**10.000**	**5000**
*Proteus vulgaris ATCC 8427*	5000	5000
*Clostridium butyricum*	NF	NF
*Clostridium butyricumATCC 860*	NF	NF
*Clostridium pasterianum*	NF	NF
*Clostridium pasterianum ATCC 6013*	NF	NF

NF **—no growth inhibition at a concentration of 100000 mg/L or 100 g/L

**Fig 11:**
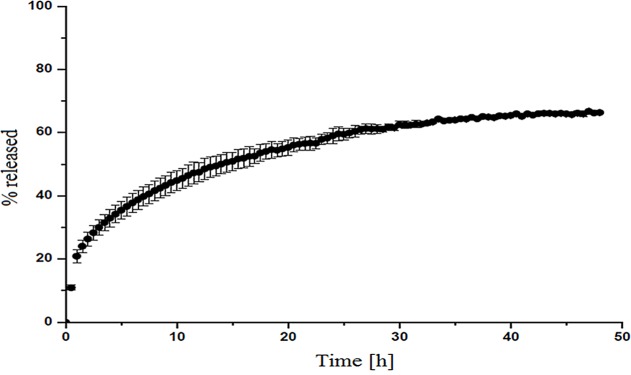
Dissolution profile of RU from RU-β-CD inclusion complex.

The dissolution profile of RU-β-CD complex is shown in Fig. 11. The dissolution studies was conducted by measurement of the concentration of released rutin from RU-β-CD complex by using UV spectroscopy. As can be seen from the figure, it was evident that the RU-β-CD complex exhibited delayed dissolution than the corresponding RU. Whole forms started with immediate release at t = 0 min, with 20% dissolved of free RU, to the dissolution up 60% at 20 h. The dissolved percentage of RU reached constant level, 60% for its complex after 25 h. The enhancement effect of RU-β-CD on the dissolution rate of RU could be connected also from the enhanced aqueous solubility of RU after the formation complex.

**Fig 12:**
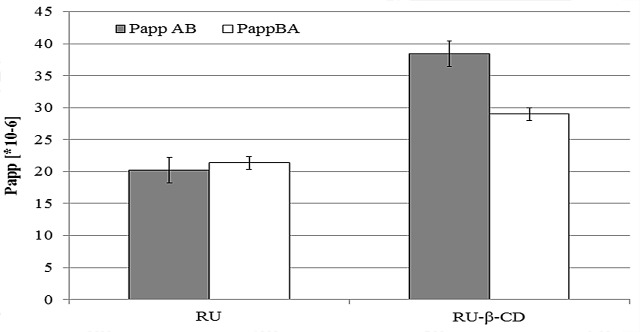
Relationship between RU and RU in inclusion complex (RU-β-CD) and permeability coefficient.

Introduction of RU to β-CD cavity resulted in increased its solubility in the donorliquid. Thus the amount of RU which penetrated through the test membrane was almost two times higher for RU-β-CD than for free RU (Fig. 12). Permeability coefficient was calculated from the following equation:

$${\text{P}}_{\text{app}}=\frac{\text{dQ / dt}}{{\text{C}}_{\text{0}}\text{ × A}}$$ (7)

where, dQ/dt is the rate of permeation of the drug across the cells, C_0_ is the donor compartment concentration at time zero and A is the area of the cell monolayer. Determination of relation P_app_(B-A)/P_app_(A-B) (<2) excluded the possibility of the participation of drug efflux inRU absorption after complexation. In each of RU forms, RU was absorbed through passive transport, as evidenced by the low values of permeability coefficients (P_app_ **=** 1.06 and P_app_ = 0.75 for RU and RU-β-CD, respectively).

## Conclusions

The study confirmed that it possible to form inclusion complexes of rutin with β-cyclodextrins by means of a grinding technique. An investigation of the identity of the RU-β-CD system permitted the determination of domains within a rutin molecule responsible for its interaction with the carrier. The possibility that the domains of rutin associated with its biological activity are involved in the stage of complex formation was rejected. It was proved that the introduction of rutin into β-cyclodextrin cavities was particularly valuable in the modification of changes of chemical stability, solubility, antibacterial activity, dissolvability. Inclusion complexes of rutin with β-cyclodextrins may therefore qualify as effective drug delivery systems for pharmaceutical application.
